# Intermolecular Interactions in Polyelectrolyte and Surfactant Complexes in Solution

**DOI:** 10.3390/polym11010051

**Published:** 2018-12-31

**Authors:** Nasreen Khan, Blair Brettmann

**Affiliations:** Materials Science and Engineering, Georgia Institute of Technology, Atlanta, GA 30332, USA; nasreen@gatech.edu

**Keywords:** polyelectrolyte, surfactant, complexes, hydrophobic, electrostatic, molecular, micelle

## Abstract

Polyelectrolytes are an important class of polymeric materials and are increasingly used in complex industrial formulations. A core use of these materials is in mixtures with surfactants, where a combination of hydrophobic and electrostatic interactions drives unique solution behavior and structure formation. In this review, we apply a molecular level perspective to the broad literature on polyelectrolyte-surfactant complexes, discussing explicitly the hydrophobic and electrostatic interaction contributions to polyelectrolyte surfactant complexes (PESCs), as well as the interplay between the two molecular interaction types. These interactions are sensitive to a variety of solution conditions, such as pH, ionic strength, mixing procedure, charge density, etc. and these parameters can readily be used to control the concentration at which structures form as well as the type of structure in the bulk solution.

## 1. Introduction

The uses for surfactants are far-reaching and have numerous industrial applications. Surfactants are used in detergents [1,2], emulsions [3,4], waste-water treatment [5], and more and their performance can be bolstered with the addition of polymers to the formulation. While surfactants and their micelles can change the surface tension, phase behavior and rheology of a solution [3,6,7], the addition of polymers, particularly charged polymers (polyelectrolytes), has been shown to enhance bulk and interfacial properties for a variety of applications [8,9,10,11,12,13,14], including cosmetics [15], perfumes [16,17], biofuel extraction [18] and oil recovery [19,20].

Mixing surfactants with neutral polymers is limited in terms of specialized functional behaviors that can be achieved by tuning of hydrophobicity. In contrast, due to the presence of charged groups, polyelectrolytes provide stronger and more tunable interactions that can be leveraged to influence properties of a given system. Surfactants have been used in combination with polyelectrolytes since the 1940s where the research specifically centered on the combination of surfactants with proteins [13,21]. Since advances in polymer chemistry allowed various synthetic polyelectrolytes to be prepared, new combinations of these charged polymers and surfactants have been explored for their resultant behavior. While polyelectrolyte and surfactant complexes (PESCs) exhibit various phenomena in the bulk, at the air-water interface, and at solid interfaces, the unique behavior is driven primarily by intermolecular interactions, both electrostatic and hydrophobic. These interactions can also be exploited by varying the attributes of the polyelectrolytes such as charge density, molecular weight, and functional groups to control properties when formulating with ionic surfactants.

**Figure 1:**
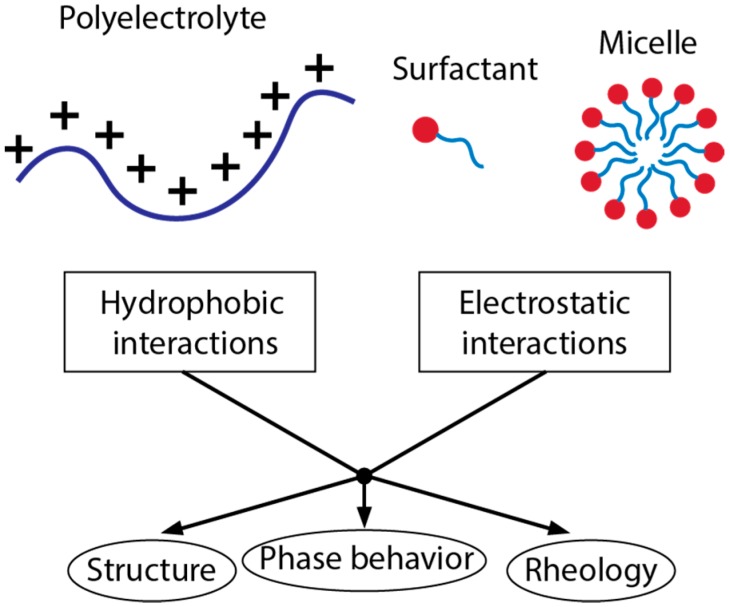
Schematic of how molecular interactions of polyelectrolyte and surfactant and surfactant micelle systems lead to structure, phase behavior, and rheological properties.

The study of PESC behavior can be separated into four conceptual parts: (1) their binding mechanisms (2) the structures that are formed (3) the phase behavior and (4) how these affect their physical behavior in terms of rheology. This review will address and analyze bulk behavior of PESC systems through the understanding of their molecular interactions and how those impact the structure, phase behavior and rheology (Figure 1). This fundamental behavior is critical for rational design of PESC formulations for industrial applications and can open the space for design of new products with advanced functionality.

## 2. Intermolecular Interactions

The performance and behavior of surfactant-polyelectrolyte systems start with the underlying intermolecular interactions. Two governing interaction types are hydrophobic, where molecules attract due to low compatibility with water and electrostatic, where the two components attract (opposite charge) or repel (like charge) due to the presence of charges. By choosing ionic surfactants and pairing them with polyelectrolytes, formulations can make use of hydrophobic, electrostatic, or a combination of both interactions.

The interactions of polymers and surfactants can be understood through their association behavior. While this is typically understood through the critical micelle concentration (CMC) for surfactants alone [1,4,6], adding a polymer or a polyelectrolyte introduces a critical aggregation concentration (CAC), or the point where the polyelectrolyte associates with the surfactant to a measurable degree [14,22]. At this point, aggregates of surfactant form that lead to micelle-like structures decorated with the polymer chain. The CAC in polymer-surfactant solutions occurs at lower surfactant concentrations than the CMC, which is the point where free surfactant molecules associate with each other to form micelles. The presence of the polymer chains makes it more energetically favorable for micelles to form [23] at low concentrations because the surfactant molecules associate with the polymer chain until a structure is formed where the polymer wraps around the micelles [23,24]. At sufficiently high surfactant concentrations, when the chain becomes saturated, micelles form from free surfactant in solution and this is the CMC [25,26,27,28]. The drivers behind these different transitions are molecular interactions, specifically hydrophobic and electrostatic. Here, we review the interaction types, factors that affect them and how they compete in driving PESC behavior.

### 2.1. Hydrophobic

Hydrophobic interactions are driven by the repulsion between a non-polar solute, such as hydrocarbons, and the surrounding polar solvent, usually water. Water molecules are thought to behave as a network of polar molecules in which the hydrogen bonding between molecules excludes other solutes [29,30,31]. Consequently, hydrocarbons in particular have low solubility and prefer to aggregate with non-polar molecules. These interactions are understood from the solubility of a non-polar species in water or a water mixture [32]. The ratio of solubilities of a solute in two different solvents can be used to calculate the free energy difference of two solvents [33,34,35], which gives the transfer free energy between two solvents, as seen in Equation (1):

(1) $$-RTln\left(\frac{s1}{s2}\right)=\Delta G_{1}^{*}-\Delta G_{2}^{*}$$

where *R* is 8.314 J/(mol*K), T is temperature, *s*_1_ is the solubility of the solute in solvent 1, *s*_2_ is the solubility of the solute in solvent 2, and Δ*G*_1_* − Δ*G*_2_* is the transfer free-energy difference between two solvents. This transfer free energy between water (Δ*G*_w_*) and a reference solvent (Δ*G*_r_*) is defined as the hydrophobicity (HY) (Equation (2)). The reference solvent is often ethanol but other solvents such as dioxane are also used [30,35].

(2) $$HY=\Delta G_{w}^{*}-\Delta G_{r}^{*}$$

Nozaki and Tanford in 1971 determined the values of the free energy of transfer of amino acids with various side chains from an organic solvent (ethanol or dioxane) to water. From this they reasoned that certain functional groups are more or less hydrophobic [35]. This thermodynamic treatment of hydrophobicity can be used to calculate the hydrophobic molecular contributions of surfactant micelle and polymer-surfactant aggregate formation from water [23,32].

**Figure 2:**
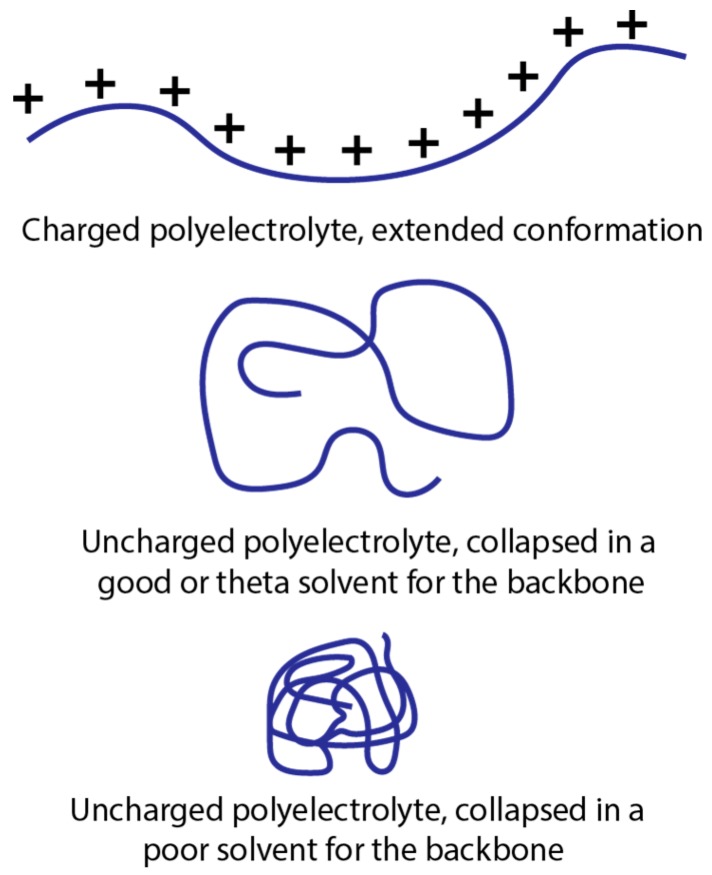
Schematic of charged polyelectrolyte conformation in good, theta, or poor solvents.

The hydrophobicity of polyelectrolytes can be measured by determining the free energy of transfer into a non-polar solvent or by their specific heat in various solvents [32,36,37]. Charged polyelectrolytes are water soluble and stay in an extended chain conformation (rigid rod) in an aqueous or polar solvent due to the favorable interactions between the charged monomers and water and repulsion between neighboring charged monomers [36]. In an uncharged state, the polyelectrolytes collapse with the degree of collapse depending on the hydrophobicity of the uncharged polymer backbone. In a good or theta solvent, the polyelectrolyte will be in random coil conformation, which is less extended than the polyelectrolyte. In a poor solvent, such as with a hydrophobic backbone in water, it will be in a collapsed conformation (Figure 2). The thermodynamic change from extended to coil state is driven significantly by hydrophobic interactions [32,36,37]. 

The strengths of the hydrophobic interactions are sensitive to many material and solution properties, and these can be used to design a system or as tuning parameters for stimuli-responsive materials. It is outside the scope of this review to cover all factors affecting hydrophobic interaction strength, but we will highlight three key parameters: temperature, co-solvents/co-solutes, and alkane chain length.

The strength of the hydrophobic interactions is temperature dependent, given that both enthalpic and entropic contributions play a role at various temperature ranges [30,32,38,39]. An increase in temperature decreases the adsorption of ionic surfactants to the oppositely charged polyelectrolytes or colloids while increasing adsorption of non-ionic ones because hydrophobic interactions increase with increasing temperature [3,40,41]. The decrease in hydration of hydrophilic groups that occurs as the temperature increases favors micellization due to the disruption of the water structure and hydrogen bonding at higher temperatures. At the same time, the micelles form through the hydrophobic moieties of surfactants and polyelectrolytes [3,40,42]. This results in an initial CMC decrease with temperature and a CMC increase with further increase in temperature [3,40]. The CAC of neutral polymer-surfactant complexes decreases with increasing temperature [40,42] due to a decrease in hydrophilicity with increased temperature, as demonstrated for polyethylene oxide (PEO) with the cationic surfactant hexadecyltrimethylammonium chloride (HTAC). For highly-charged polyelectrolyte systems, there is an influence of temperature, however the system is more strongly influenced by the electrostatic interactions, which have only a minor temperature dependence [3,37,43,44,45].

Solvents other than water can also produce solvophobic effects that differ in strength from the hydrophobicity. While the majority of PESC studies are conducted in aqueous solution, a few have been done in organic solvents [22,39] or introduce additional organic co-solutes or co-solvents such as alcohols or alkanes which can increase or decrease the driving force for complexation between polyelectrolyte and surfactant. Conformation of the polymer and surfactant changes in a given solvent depending on the solvophobicity, as was discussed previously (Figure 2) [22,32,37,39,46,47]. These conformational changes lead to changes in the structure of the micelles formed [47,48,49], the number of surfactant molecules in a micelle (mean aggregation number) [22,46,47,50], the viscosity [22,32,39,47], and the phase behavior in terms of insoluble or soluble complexes [32,39,51]. Consequently, non-polar solutes in a non-polar solvent are more molecularly and energetically compatible, similarly with polar solutes in a polar solvent [47]. Therefore adding co-solutes or co-solvents can tune desired micelle, phase and other properties.

Increasing the number of alkyl groups in an alkane chain in the surfactant tail increases the hydrophobicity due to the low solubility of the non-polar alkyl group in the aqueous solution. The free energy required to transfer alkanes into water increases with increasing number of methylene groups in linear N-alkanes [30,31,33]. Because hydrophobicity increases with the number of alkanes, increasing the chain length for surfactants complexed to a polyelectrolyte reduces the CMC and CAC, favorably lowering the free energy of both transitions and leading to stronger interactions within micelles [22].

### 2.2. Electrostatic

Electrostatic interactions are described by Coulomb’s law (Equation (3)), which calculates the magnitude of the force (*F*) created by the repulsion or attraction of two charged points (*q*_1_ and *q*_2_) over the squared distance (*r*) between them. The Coulomb’s constant (*K*_e_) accounts for the dielectric permittivity of the medium [52], which, in the case of typical polyelectrolyte and surfactant systems, is that of water [53].

(3) $$F=\frac{K_{e}q_{1}q_{2}}{r^{2}}$$

The local charge density and the local counterion concentration can be taken into account by the Debye-Hückel model to calculate the Debye length (*κ*^−1^).

(4) $$\kappa^{-1}=\sqrt{\frac{\varepsilon_{r}\varepsilon_{0}k_{B}T}{2N_{A}e^{2}I}}$$

where *ε*_r_ is the dielectric constant, *ε*_0_ is the permittivity of free space, *k*_B_ is the Boltzmann constant, *T* is the temperature, *N*_A_ is Avagadro’s number, *e* is the elementary charge and *I* is the ionic strength of the electrolyte solution. The Debye length is defined as the distance from a charge surrounded by other point charges that the interaction potential is screened. This is accepted for simple salts, but for polyelectrolytes the model is limited because it treats the ions as a point charge, whereas for polyelectrolytes the charges are distributed along a chain and thus are not point charges [53,54]. Charged functional groups are periodic along the backbone or on a pendant off of the main chain when dissociated. Despite the imperfection of the model, the Debye length is commonly used in theoretical treatments to model the interaction between charged chain segments [23,53]. The Bjerrum length (*λ*_B_) is also used to describe electrostatic interactions and is defined as the distance between two charges at which the interaction strength is comparable in magnitude to the thermal energy, *k*_B_*T*. The expression for *λ*_B_ is:

(5) $$\lambda_{B}=\frac{e^{2}}{4\pi \varepsilon_{0}\varepsilon_{r}k_{B}T}$$

This is commonly used to describe the strength of the electrostatic interaction in polyelectrolytes [52,53,55].

Electrostatic interactions of polyelectrolytes are also highly sensitive to their oppositely charged counterions, which can be free in solution or tightly associated to the chain (condensed), effectively neutralizing those charges and decreasing the overall apparent polymer charge. Manning developed a theory for counterion condensation using the Debye-Hückel approximation. Instead of treating polyelectrolytes as point charges, the model represents the polyelectrolyte chain as an infinite line charge with a given linear charge density, *β*:

(6) $$\beta =z_{p}e/b$$

where *z_p_* is the valence of the charged groups on the polymer, *e* is the elementary charge and *b* is the distance between charges on the polymer. It has been shown that the statistical mechanical phase integral for such an infinite line charge diverges at high charge densities. This essentially means that the system is unstable at linear charge densities greater than a critical value, *β* > *β_crit_*. 

Manning developed the model in terms of the charge parameter, *ξ*, for polyelectrolytes in solution.

(7) $$\xi =\frac{e^{2}}{\varepsilon KTb}=\frac{\lambda_{B}}{b}$$

For counterion *i*, the system is unstable for values of $\xi \geq {\left|z_{i}z_{p}\right|}^{-1}=\xi_{\mathrm{crit}}$, or for monovalent ions, ξ≥1, where *z*_i_ is the valence of the counterion. As the system is unstable for values of *ξ* > *ξ*_crit_, counterions will “condense”, or associate closely to the polymer chain until *ξ* approaches *ξ*_crit_ (1 for systems of monovalent ions). Since *ξ* is a ratio of the Bjerrum length to the average charge spacing, the critical charge spacing for a system with monovalent ions is equal to the Bjerrum length and for multivalent counterions is equal to *λ*_B_*/z_i_*. Since only a fraction of the counterions condense onto the chain, the remainder are mobile [56]. The mean-field theory models discussed here are for idealized systems of strong polyelectrolytes with point ions and they neglect more complex effects such as specific ion interactions, dipole-dipole interactions, and counterion-induced correlations, which often add attractive interactions to the system. However, they still provide valuable phenomenological insights into polyelectrolyte behavior.

Manning focused on theoretical descriptions of polyelectrolytes with their counterions, but the interaction of ionic surfactants with oppositely charged polyelectrolytes is a special type of interaction with “counterions”, as the surfactant acts as a counterion, but each molecule is more complex than a small, inorganic ion. Oppositely charged polyelectrolytes and surfactant head groups will associate in solution. The association is thought to be driven primarily by the entropic gains when multiple small counterions are released as the two larger molecules associate, although enthalpic contributions are also present [57,58,59]. There is theoretical similarity to polyelectrolyte complex coacervate formation [41,48,53,60,61,62,63,64], but in practical applications of polyelectrolyte-surfactant complexes, the hydrophobic interactions also play a large role and the balance of the two interaction types is of great importance.

The most important factors that affect electrostatic behavior are ionic strength [8,53], pH [65,66,67], and counterion valency [8,53,68,69]. Because polyelectrolytes have charged moieties along the length of the polymer chain, the electrostatic interaction is significant when they are fully charged, [26,27,70,71] and the effects from electrostatic interactions compete with the hydrophobic effects in driving morphology and phase behavior [47,72,73,74]. Due to the dependence of behavior on easy-to-adjust parameters, such as ionic strength and pH, PESCs are attractive as stimuli-responsive materials.

The solution ionic strength strongly influences the behavior of PESCs, as the salt background can reduce the strength of electrostatic interactions. Increasing the concentration of salt weakens the coulombic potential energy between ions [52]. At low salt concentrations, the strong electrostatic interactions drive significant attraction between a charged surfactant head group and an oppositely charged polyelectrolyte chain. However, at high salt concentrations, ionic surfactants have weaker interactions with the polyelectrolytes and behave more like neutral polymers in solution [8,71,75,76]. Therefore, both the CAC and CMC occur at higher surfactant/polyelectrolyte concentrations at high ionic strengths [45,77,78].

Electrostatic interactions between charged surfactants and polyelectrolytes can be manipulated by the pH when the polyelectrolyte and/or surfactant are weak acids or weak bases. If the pH is not sufficiently acidic or basic, which depends on the functional group, the dissociation of the charge may be reduced or eliminated, resulting in a polymer that is essentially neutral and eliminating electrostatic interactions. This has been seen for weak polyelectrolytes including poly(methacrylic acid) (PMA) [22,37,79], polyacrylic acid (PAA) [22,32,37,80], poly(4-vinylpyridine *N*-oxide) (PVPNO) [65,66], and a maleic acid-co-polymer [37,76].

When there are sufficient surfactant molecules to have a stoichiometric match with the charges on the polyelectrolyte chain, the associated complex is essentially neutral and will phase-separate out of water due to low solubility in water [9,14,58]. In 1977, Goddard et al. studied the association of the anionic surfactant sodium dodecyl sulfate (SDS) and a cationic derivative of hydroxyethyl cellulose [13,14,70]. Through these initial studies, it was observed that the maximum precipitation between polyelectrolytes and surfactants occurs at the charge neutralization point, which occurs after the CAC.

Increasing the charge density, or how many charged monomer units are on a polyelectrolyte, will increase the strength of the electrostatic interactions because the Coulomb’s interaction is proportional to the number of charges. The larger the charge density, the larger the contribution of the electrostatic effects in the system, leading to surfactant and polyelectrolyte association at lower surfactant concentrations, shifting the CAC lower [9,58,70,81]. Similarly, increasing the number of charged groups on the surfactant, for example through use of Gemini surfactants, which have two charged heads and two alkyl tails, decreases both the CMC and CAC by several orders of magnitude beyond typical values for surfactants and polymer combinations [14,82,83,84].

### 2.3. Balance of Electrostatic and Hydrophobic Interactions

Both electrostatic and hydrophobic interactions contribute to polyelectrolyte and surfactant associations. Depending on the system, the hydrophobic or the electrostatic interactions can play a larger role. Together both effects depend on the temperature, pH, salt concentration, hydrocarbon moieties within the main chain of the polyelectrolyte or in the pendant, as well as solvent or co-solute [11,39,53,59].

**Figure 3:**
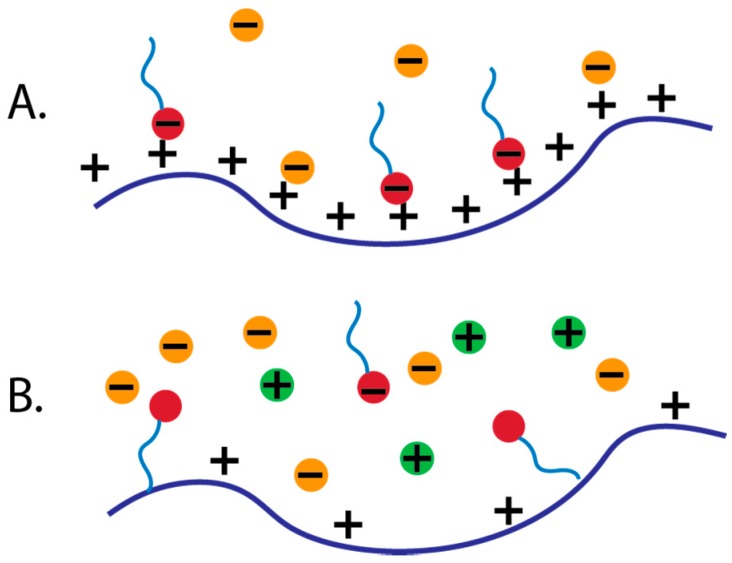
Depiction of binding of surfactant to polyelectrolyte chain. (**A**) without the presence of added salt (**B**) in the presence of added salt.

As discussed previously, with oppositely charged polyelectrolyte and surfactant combinations high concentrations of salt decrease the electrostatic interactions, which leads to interesting phase behavior. With no added salt, the entropy increase from the release of counterions into the bulk after complexation leads to electrostatically-driven associative phase separation of the surfactant head and polyelectrolyte (Figure 3A) [47,59,63,85]. As the salt concentration is increased and charge screening is present, the electrostatic interactions weaken, and at high concentrations of salt, the polyelectrolyte and surfactant are effectively neutral and their association is more sensitive to hydrophobic interactions of the surfactant tail and polyelectrolyte backbone (Figure 3B) [59]. In a case where the polyelectrolyte is more polar and hydrophilic, hydrophobic-driven phase separation occurs only at high salt concentration, while more nonpolar polyelectrolytes may show phase separation driven by hydrophobic interactions at a wider range of salt concentrations [9,46,47].

Polyelectrolytes can be modified or prepared with alkyl chain blocks to add hydrophobic moieties to their structure and utilize both electrostatic and hydrophobic contributions for binding, structure, and phase behavior of PESCs. Delisavva, et al. demonstrated that when a block co-polymer with a cationic block and a neutral block, poly(2-vinylpyridine)-*block*-poly(ethylene oxide) (P(2-VP)-PEO) is combined with a Gemini surfactant, the hydrophobic nature of the double surfactant tail plays the more dominant role in the formation of the surfactant bilayer because of the large hydrophobic regions and increased flexibility of both the surfactant and polyelectrolyte structures [82]. Bai et al. studied the interactions between a newly-synthesized hydrophobically modified dextran polyelectrolyte, D40OCT30, and found a decrease in total interaction enthalpy and strong association with surfactants with increasing D40OCT30 concentration. The total interaction enthalpy has contributions from the hydrophobic interactions between alkyl side chains and surfactant tails, as well as some electrostatic interactions from the head group and polyelectrolyte. The increase of the enthalpy, then, implies that both electrostatic and hydrophobic interactions of the polyelectrolyte with SDS contribute to the formation of complexes, although the two effects cannot be separated in this type of study [59]. Furthermore, Liu et al. found through simulations that not only do the resulting shape and size of PESC aggregates change, but a larger number of surfactants adsorb to a block polyelectrolyte when alkyl chain blocks are added to the copolymer [72].

The aggregation number of polyelectrolyte-surfactant aggregates has also been found to depend on the polyelectrolyte charge. For a copolymer with one methyl, the CAC was reduced at high degrees of dissociation (high polyelectrolyte charge) for the copolymer. This is because the polyelectrolyte binds to the surfactant through electrostatic interactions between the charged surfactant head and the highly charged polyelectrolyte copolymer, even when there are fewer charged groups and a larger spacing between them. The decrease in the CAC was even stronger for a copolymer with four methyls, as it displayed both hydrophobic and electrostatic contributions to association [86]. 

In polyelectrolyte and charged surfactant systems, both electrostatic and hydrophobic interactions contribute to the binding, structure, and phase behavior of PESC systems. The electrostatic contributions can be readily manipulated by the addition of salt or changes in pH and can force the polyelectrolyte and surfactant to behave more like a neutral polymer, where hydrophobic forces come more into play. In this case, the binding is between the surfactant tail and polyelectrolyte [65]. For charged systems, electrostatic interactions are responsible for the initial binding and adsorption of charged surfactants to polyelectrolytes because these are strong long range interactions. Hydrophobic interactions then help the aggregation and formation of micelle-like structures though the interactions between nearby surfactant tails. Additionally, hydrophobic interactions often improve aggregation and complexation when polyelectrolytes are modified to include hydrophobic moieties because the surfactant tails can then adsorb and aggregate along the chain at these hydrophobic portions, as well as initially electrostatically at their heads [59,72].

## 3. Interactions of Polyelectrolytes and Surfactants in the Bulk Solution

Polyelectrolyte and surfactant complexes are known to exist and interact concurrently in bulk solution and at the interface between air and solution (air-water interface) given the amphiphilic nature of surfactants. This section focuses on the complexes that form in the bulk solution, first discussing the formation of complexes given the binding mechanisms between polyelectrolyte chains and individual surfactants, then the structures and aggregates that are formed. We also discuss the experimental factors that affect complexation.

### 3.1. Formation of Complexes

#### 3.1.1. Binding Mechanisms—Cooperative, and Non-Cooperative

**Figure 4:**
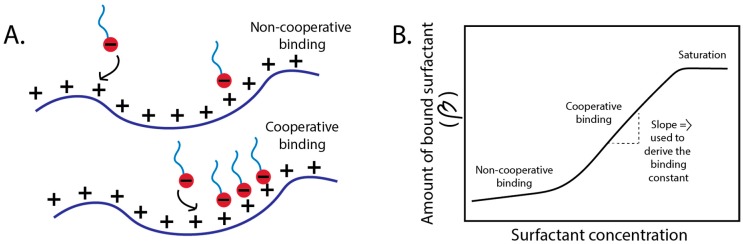
(**A**) Non-cooperative and cooperative binding of surfactant to polyelectrolyte (**B**) Illustration of the binding isotherm: degree of binding (β) versus surfactant concentration.

Binding of polyelectrolytes and surfactants is the first stage in forming complexes and phase separated structures in solution and can occur through two main mechanisms. These mechanisms are cooperative and non-cooperative binding. Non-cooperative binding of a surfactant to a polyelectrolyte occurs first and is where the adjacent binding sites are not already filled by surfactant molecules. Cooperative binding occurs after the adjacent sites are already filled (Figure 4A) [63,87].

Binding isotherms depict the degree of binding (β), which is the ratio of the concentration of bound surfactant to the concentration of polyion charges. Therefore as β approaches 1, the polyelectrolyte is fully saturated with surfactant [23,87,88,89]. Non-cooperative binding occurs at very low amounts of surfactant added to the polyelectrolyte (dilute), before cooperative binding and can be difficult to resolve experimentally [87,90]. Cooperative binding is identified by a sigmoidal curve and begins in the early stages of binding, where the system is still in the dilute regime, but the surfactant concentration is higher than for non-cooperative binding. The binding amount in this regime increases linearly with increasing concentration of surfactant and the binding constant (Ku) in this regime can be derived from the slope of this linear portion (Figure 4B) [76,91]. The binding constants give an indication of the strength of the interactions between molecules [22,78]. When the system reaches the CAC or CMC, plateaus or inflections are seen in the binding isotherm [22,58,71,76,92].

From binding isotherms, thermodynamic values of the free energy, entropy, and enthalpy are derived for binding behavior, aggregation, and overall complexation [13,23]. By varying the type of polyelectrolyte or surfactant, the hydrophobic moieties [59], or the surfactant tail length [84,92,93], the thermodynamic values can be compared to assess which systems have the most favorable free energy and whether the complexation is entropically or enthalpically favored. 

Important insights into the binding mechanism of polyelectrolytes with surfactants have been made through studies of neutral polymers with surfactants. In an early study, Nagarajan developed equations to describe the thermodynamics of micellization of anionic SDS or neutral Triton X with the neutral polymer PEO, and the dependence of the micellization process on surfactant concentration for non-ionic polymers. The free energies of micellization were determined for the surfactant alone, the surfactant and polymer, and the polymer with two competing surfactants. This treatment took into account the hydrophobicity of the surfactant tail and the electrostatic contribution for the anionic head of SDS [34]. By comparing the theoretical thermodynamics and experimental data for binding of PEO to SDS versus Triton X (a non-ionic surfactant), it was shown that the binding of SDS to PEO is more favorable. For Triton X, which has a neutral bulky head, free micelles are formed instead of complexing with the PEO [23]. This study agrees with other studies that show that neutral surfactants form only weak associations with neutral polymers compared to charged surfactants [14,25,94] and that weak interactions of polymers with cationic surfactants increases the hydrophobicity of the surfactant tail [95]. 

Barbosa et al. examined the binding isotherms of PEO and SDS in the presence of ionic salts. Increasing the surfactant concentration decreased the observed enthalpy of binding, but increasing the salt concentration did not have a significant effect on the binding or the thermodynamics of the system except with the complex ionic salt Na_2_[Fe(CN)_5_NO]. Enthalpic titration curves were similar for various PEO-SDS simple salt systems, indicating that for this non-ionic polymer, the interaction behavior is primarily due to hydrophobic interactions between the surfactant tail and PEO, which is to be expected since the polymer is not charged and thus cannot interact through electrostatic interactions [96].

For PESCs, binding between charged polyelectrolytes and surfactants occurs at very low surfactant concentrations and is primarily electrostatic. In a salt-free system, the binding can be slightly exothermic and it becomes more endothermic with increasing salt concentrations [77]. The binding of a charged polyelectrolyte to a charged surfactant has been found to be entropically-driven [72,77], as is also the case between oppositely charged polyelectrolytes [41]. The overall change in enthalpy for the process is generally endothermic and also gives insight to how individual factors contribute to the overall thermodynamics of binding. The binding isotherms are polyelectrolyte and surfactant dependent, where more hydrophobic charged surfactants will have larger cooperative binding parameters with more hydrophobic polyelectrolytes [92,97]. However, they will also have larger cooperative binding parameters when the polyelectrolyte has a higher charge density [98] or at higher salt concentrations [77,78,92,97]. 

In the case where the polyelectrolyte is fully charged and no salt is present, the polyelectrolyte is in a rigid extended state. The electrostatic binding of the surfactant decreases the number of charged groups on the polyelectrolyte, which causes the polyelectrolyte to transition from a rigid rod to a coil before aggregation and micelle-like formation occurs, typically resulting in a string-of-pearl configuration for the PESC, as will be discussed further in Section 3.1.3 [72,99,100].

#### 3.1.2. Formation of Micelle-Like Structures

**Figure 5:**
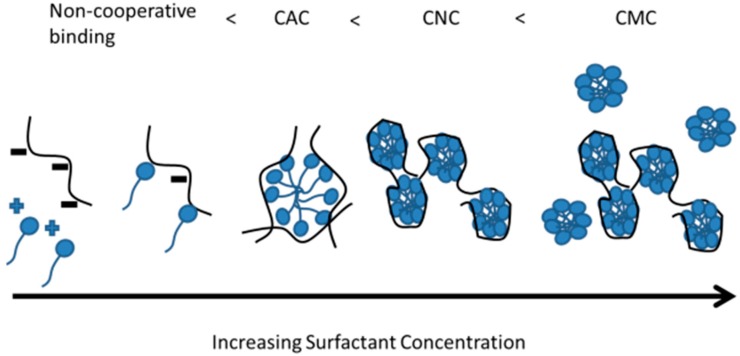
Progression of micelle-like structure formation with increasing surfactant concentration.

Beyond the binding of the polyelectrolytes to surfactants, it is also important to understand the progression from solution to aggregates to micelles as the concentration of the surfactant is increased. As illustrated in Figure 5, initially, in a salt free system and at low surfactant concentrations, the oppositely charged surfactant head electrostatically attracts to a charged monomer unit on the polyelectrolyte. With increasing surfactant concentration, the system reaches the CAC and the polyelectrolyte forms a micelle-like aggregate with the surfactant tails forming a hydrophobic core and the charged surfactant heads interfacing with the polyelectrolyte [23,39]. A single polyelectrolyte can interface with many micelles or micelle aggregates or a single micelle can interact with multiple chains [59,79]. When enough surfactant is present to reach the charge-neutralization point (at or near a 1:1 molar charge ratio), all the free charges on the polyelectrolyte are neutralized by the charged surfactant heads and this is the charge neutralization concentration (CNC) [77]. This leads to the formation of precipitates, ordered structures, and gels that phase separate [9,39,59,101,102,103]. Increasing the surfactant concentration further allows the hydrophobic surfactant tails to interface with the now neutral precipitate and the charged heads to interact with water. This eventually leads to re-solubilization of the complex and the formation of micelles outside of the complex (CMC) [26,59,63,68,70]. Therefore, the concentration of the surfactant will dictate the phase behavior after the charge-match point.

**Figure 6:**
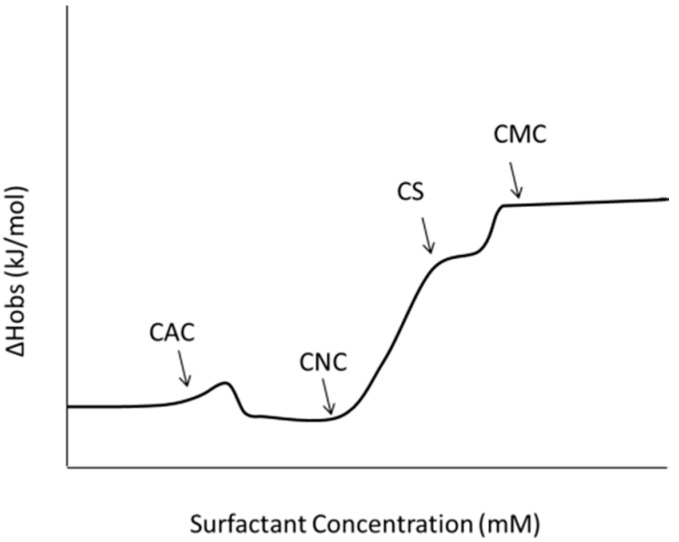
General depiction of observed enthalpy versus increasing surfactant concentration for polyelectrolyte and surfactant systems.

The thermodynamics of the progression of complexation and micelle formation in polyelectrolyte-surfactant systems have also been examined in detail. The overall observed enthalpy change with the addition of surfactant to the polyelectrolyte is seen to be positive and endothermic, further confirming that binding and aggregation of surfactant with polyelectrolyte is entropically driven from the release of counterions [58,59,63,65,77,96,98]. As illustrated in Figure 6, at low concentrations of added surfactant there is an initial plateau indicating the non-cooperative binding that occurs first in PESC formation, followed by an inflection point where cooperative binding begins, indicating that the CAC has been reached before a cooperativity peak where favorable hydrophobic interactions between adjacent surfactant tails start to form micelle-like structures [63]. Finally, a drop in enthalpy occurs after the cooperative peak, where electrostatic interactions are leading to neutralization. Then the enthalpy plateaus until it reaches the charge neutralization (CNC), after which another inflection point is seen. The enthalpy then increases until a saturation point is reached (CS) where the polyelectrolyte chains are fully saturated by surfactant in micelle-like aggregates [58,85] and finally free micelle formation (CMC) occurs. The saturation point can be mistaken as the CMC and is sometimes difficult to identify [58]. The CS point occurs after the charges on the polyelectrolyte chain have been neutralized, but the PESCs continue to grow due to hydrophobic interactions between the surfactant tails with increasing concentration [58,85]. Near this point, the charge (positive or negative) of the complex changes to that of the surfactant [85].

Nizri et al. studied the mechanism of polyelectrolyte and surfactant binding and formation of complex aggregates and nanoparticles. They saw that for an SDS- cationic polydiallyldimethylammonium chloride (PDADMAC) system, the electrostatic contributions were not alone in driving the formation of nanoparticles. With increasing salt concentration (more charge screening), the cooperative attachment of a surfactant to a polyelectrolyte site increased, as was seen from increased observed cooperative binding parameters and an increase in observed enthalpy from isothermal calorimetry (ITC) data. These results indicated that the formation of SDS-PDADMAC aggregates was driven by a combination of electrostatic and hydrophobic interactions [77]. The influence and importance of hydrophobic interactions on binding and aggregation between surfactant and polyelectrolyte has been proven to be present even as electrostatic interactions and hydrophobic interactions for various systems are altered by changing temperature [45], pH [58], increasing of alkyl chain length [92] or the addition of salt [65,83]. Increasing hydrophobicity tends to increase binding and aggregation behavior in polyelectrolyte systems in regimes where electrostatic interactions are not very large [104].

The individual transitions, such as CAC, CNC, CS, CMC are influenced by changes in electrostatic or hydrophobic interactions (additional salt, pH, temperature, added hydrophobic regions). CAC in particular shifts to lower values for more favorable interactions with increasing electrostatic and hydrophobic contributions. This is seen in examples with PDADMAC-SDS [77], (PVPNO)-SDS, [65], PAA-C_14_TAB [80], and polyethyleneimines (PEI)-SDS [58], to mention a few.

#### 3.1.3. Types of Structures Formed and PE-Micelle Interactions

**Figure 7:**
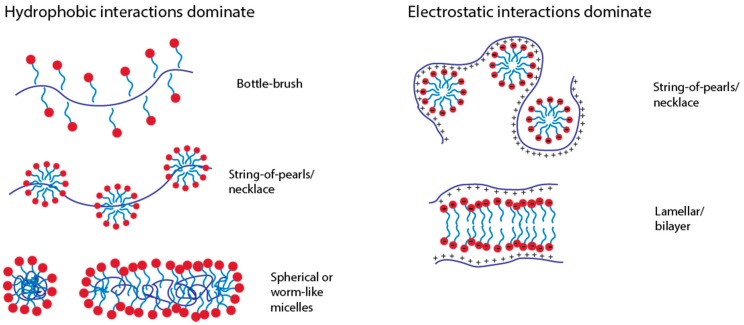
Typical micellar or micelle-like structures formed with polyelectrolytes.

Surfactants in solution form various micellar structures including spherical, cylindrical, and bilayer, as seen in Figure 7 [3,6]. The forms are largely a result of the size and shape of the surfactant and the length of the alkyl tail [6,105]. Surfactants form micelles in solution and can go through a sphere-to-rod transition with the addition of salt, which screens the charged headgroups, reducing the resulting curvature of the micelles and allowing growth in one direction through hydrophobic interactions between the tails, leading to a more rod-like shape [106]. The presence of oppositely charged polyelectrolytes in solution with surfactant molecules assists in the formation of various micellar and micelle-polyelectrolyte structures in both a liquid and solid phase. The overall shape and curvature that the surfactant micelles take on, along with the association to polyelectrolytes gives rise to structures that include string-of-pearls and wormlike micelles with polyelectrolytes wrapped around micelles [100,107,108].

The type of structure that forms in the bulk depends on the electrostatic contributions from the charged species, the pH, the hydrophobicity of the surfactant tail and polymer chains [100], the molecular geometry between the surfactant micelle and polyelectrolyte [85,100,105], the charge density [109] and the solvent [39,100]. In addition to the surfactant micelle shape, the structure is further complicated by the polyelectrolyte conformation, or whether it is in a rigid rod or coil configuration at the solution conditions. The rigid-rod to coil transitions of the charged polyelectrolytes can occur as the charges are neutralized by the opposite charge on the surfactant [109], or at a pH where the polyelectrolyte is minimally charged, changing the final shape of the aggregate [100,106].

At low polyelectrolyte charge, the system will be dominated by hydrophobic interactions and behave the same as a neutral polymer mixed with a surfactant. At low concentrations for neutral polymers such as PEO, the hydrophobic tail associates along the polymer (Figure 7) [13,96,106,110]. The resulting micelle-like aggregates that form are charged due to the ionic surfactant head since the tail associates with the polymer [23,39,58,59]. The result is typically a string-of-pearls or necklace structure at moderate surfactant concentrations and hydrophobic interactions [23,96,100]. This is commonly observed with charged surfactants and neutral polymers, as well as polyelectrolytes with low charge densities [23,106,109]. The hydrophilicity and hydrophobicity of the polymer backbone often dictates the resulting structure as well. For more hydrophilic polyelectrolytes, the complex moves from a bottle-brush to string-of-pearls/necklace to wormlike micelle with increased surfactant concentration (Figure 7). For more hydrophobic polyelectrolytes, a spherical micelle is formed at high surfactant concentrations with the polyelectrolyte chain able to penetrate into the core [106,108].

As the polyelectrolyte charge increases, the binding behavior is dictated largely by the electrostatic interactions between the charged head and the polyelectrolytes (Figure 7). The string-of-pearls/necklace, which in this case is formed by the polyelectrolyte chain attracting to the outside of the micelles, rather than the core, is favored for extended chains of charged polyelectrolytes, and the degree to which the string-of-pearls stretches out depends on the flexibility of the polyelectrolyte [100]. In the case of highly stiff polyelectrolytes and block copolymers, a layered lamellar-like structure can form [72,111].

PESCs also form solid precipitates in solution, which have structures of varying morphologies [105,105,112,113]. Numerous studies discuss the structure and formation of well-ordered precipitates or colloidal nanoparticles that form from PESCs [39,105,107,112,113,114]. Antionetti et al. examined PAA and poly(styrene sulfonate) (PSS) with alkyltrimethylammonium surfactants of varying lengths at a 1:1 stoichiometric ratio. When the alkyl chain length of the surfactant was increased by only a few carbons, the resulting mesophase structure changed from disordered to ordered lamellar structures with alternating ionic and hydrophobic surfactant tail lamellar layers. Further increasing the alkyl chain length drove a transition from a lamellar layer, to a “mattress” structure in which undulating divets formed in the layers due to the physical packing differences between the ionic phase and alkyl hydrophobic phase. A further increase in alkyl chain length led to perforations in the lamellar layers [105,112,113]. This is thought to be because surfactants with longer chains show more ordered packing [39,105] and geometric restrictions of the surfactant [105].

Deviating from stoichiometric charge match, the resulting precipitates can take the form of bulk aggregates or stabilized particles, depending on whether one component is present in sufficient excess. The structures can re-solubilize with increasing surfactant concentration, which leads to dilution from thread-like or lace-like structures to liquid crystalline aggregates to spheroidal micelles [107]. The primary factors differentiating between the different precipitate structures in all these studies are the properties of a given polyelectrolyte (charge density, hydrophobicity, tacticity), the stoichiometry between the polyelectrolyte and surfactant charged groups, and the length of the surfactant tail or hydrophobic blocks in a block co-polyelectrolyte [105]. 

Of industrial importance in these systems is how to maintain single phase behavior (prevent precipitation) in ways other than increasing the concentration beyond 1:1 stoichiometry. Adding non-polar hydrophobic co-solutes and a higher concentration of salt [46,47,96] has been shown to maintain one phase where behavior is associative. MacKnight et al. also noted that solid polypeptide precipitates can be solubilized in organic solvents of low polarity, such as with high concentrations of trifluoroacetic acid (TFA), even when the ions in the polymers are not dissociated [39]. However Nilsson et al. noted that the addition of octane to oppositely charged polyelectrolyte and surfactant systems had a negligible effect on the phase behavior of PAA and C_14_TAB because the combination of oppositely charged polyelectrolytes and surfactants resulted in associative phase separation where the hydrophobic interactions present between neutral complexes are sufficiently significant to stabilize this phase, whereas further addition of a hydrophobic co-solute is negligible [47].

A coacervate forms when liquid-liquid phase separation occurs due to the association of a polyelectrolyte and an oppositely charged micelle or polymer-micelle aggregate near charge-neutrality [59,85]. These can occur at medium concentrations of surfactant for some systems beyond the CNC, can be induced by dilution of precipitates with water at a constant salt concentration [9,48,57,115], or can be formed by addition of high concentrations of salt and surfactant [57].

### 3.2. Experimental Factors Affecting Complexation

In the previous section, the intermolecular interactions were introduced and the structures that form in the bulk were discussed. Here the specific experimental factors that can be controlled to utilize those interactions to alter binding, structures and phase will be discussed. Electrostatic interactions are influenced by pH, charge density, and salt concentration. Hydrophobic interactions are influenced by chain length, molecular weight or structure of the polyelectrolyte chains or surfactant tails. Other experimental parameters, such as surfactant concentration, stoichiometry, and mixing procedure also impact the formation and properties of the PESCs. In altering these parameters, changes in outputs of the resulting nanoparticles and phases such as surface charge, turbidity, and particle size are seen.

#### 3.2.1. Concentration and Stoichiometry

The concentrations of the surfactant and polyelectrolyte, as well as the stoichiometry between the two are easy experimental parameters to adjust to promote formation of desired PESC structures. These experimental parameters affect the progression of the complex from solution to aggregates to micelles. The PESC transitions from non-cooperative to cooperative binding (C < CAC) to initial formation of micelle-like aggregates (CAC) to charge neutralization and saturation (CNC and CS) and to free-micelle formation (CMC) as the surfactant concentration is increased (Figure 6). At even higher surfactant concentration, beyond saturation, resolubilization occurs [58].

**Figure 8:**
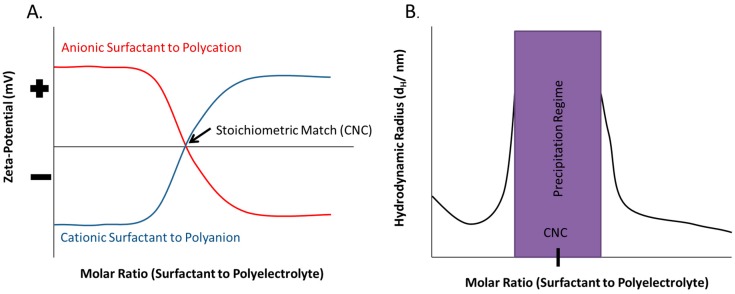
(**A**) Zeta-potential versus molar ratio of surfactant to polyelectrolyte. (**B**) Hydrodynamic radius versus molar ratio of surfactant to polyelectrolyte. Adapted from [107,108,115,116].

In addition to the progression of complex types, the particle size and charge of PESC nanoaggregates are also dependent on the stoichiometry and which component is in excess [77,107]. At low surfactant to polyelectrolyte ratios, the particles are the same charge as the polyelectrolyte. As illustrated in Figure 8A, as the molar ratio approaches a stoichiometric match, the complex approaches the charge of the surfactant and becomes neutrally charged near the CNC, and as the molar ratio is further increased to be majority surfactant, the charge continues to increase or decrease to the charge of the surfactant head [77,107,115]. At the same time, the hydrodynamic radius of the complex changes with surfactant concentration, as illustrated in Figure 8B. At low surfactant concentration, the particles are stabilized by the polyelectrolyte charges, but as the concentration of surfactant increases, the repulsion of the charged micelle-like particles decreases, removing the barrier to aggregation, and the measured size of the particles increases due to increasing aggregation. Near a stoichiometric match point, precipitates are formed and the hydrodynamic radius cannot be appropriately measured. However, at higher surfactant concentrations, at a stoichiometric ratio greater than 1, there is an increase of charged surfactant molecules in the complex, and the particles become more charged and de-aggregate, producing smaller measured particles [107,108]. For very high surfactant to polyelectrolyte concentration ratios, the bulk solution is a single stable associative phase including both polyelectrolyte, salt, and surfactant in which re-dissolution of the separated phase occurs and free micelles form in solution [68,85,115].

When the concentration of polyelectrolyte is increased, the number of charged groups available for electrostatic interaction increases and higher concentrations of surfactant are needed to reach the CNC [85]. The concentration range of surfactant where precipitation occurs is also broader for higher polyelectrolyte concentrations and results in larger diameters of the charged particles past the stoichiometric match [57,115]. The hydrodynamic radius is also larger at larger polyelectrolyte to surfactant concentration ratios [85], likely due to repulsion between excess polyelectrolyte chains bound in the complex. In terms of binding, ITC measurements have shown that increasing the polyelectrolyte concentration in the dilute regime results in only a minor binding strength decrease in terms of cooperative binding values and molar enthalpy of cooperative binding with increasing polyelectrolyte concentration. The binding interactions scale with the total number of charged sites available, but the binding per site is independent of polyelectrolyte concentration [85].

The concentration of the polyelectrolyte and surfactant and the stoichiometric ratio between the two are some of the most common parameters used to control the formation of PESC particles in the bulk, leading to transitions from soluble complexes to aggregated precipitates to nanoparticles. They also influence the resulting particle size and charge, which are critical to understand and control when preparing an industrial formulation that relies on PESCs.

#### 3.2.2. Surfactant Tail Length and Polyelectrolyte Molecular Weight

The tail length of the surfactant affects the binding interactions, the structure, and the phase behavior of a PESC, given the strong influence of chain length on hydrophobicity. The additional alkyls increase the propensity of hydrophobic interactions between polyelectrolyte and surfactant. These interactions become especially significant in the cooperative binding region [13,72,78,97]. In addition to increasing binding strength in the cooperative binding region, increasing the chain length of the surfactant lowers the CAC [84,91]. Wallin and Linse used Monte Carlo thermodynamic simulations in conjunction with experimental data to show that the increase in surfactant tail length of CTAB from an 8 to 16 carbon tail led to a decrease in the CAC/CMC ratio [84]. Numerous additional studies have been in agreement in terms of the reduction of CAC and CMC with increasing tail length, including for both cationic and anionic surfactants. This is because the binding and the complexation process is favorable in terms of free energy and cooperative binding [22,78,87,92].

**Figure 9:**
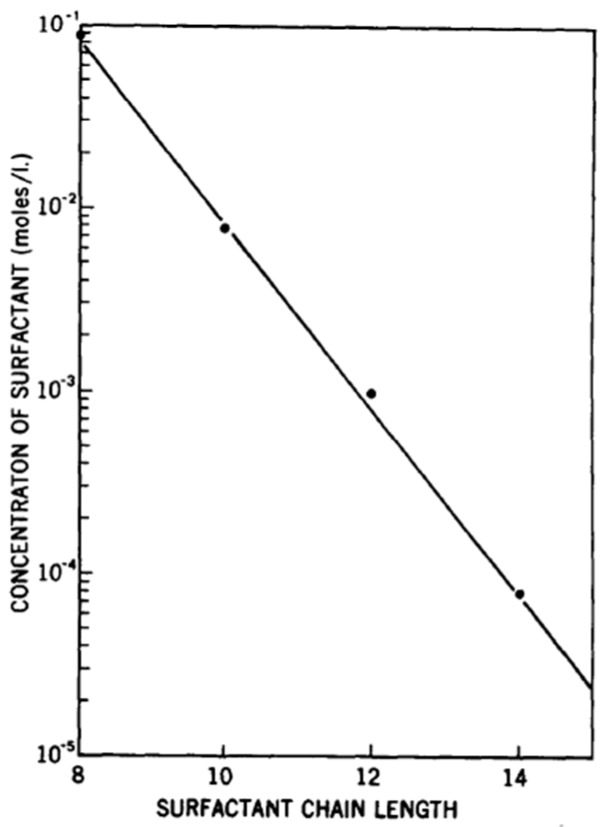
Concentration of SDS surfactant needed to maintain a precipitated complex versus surfactant chain length. Reprinted with permission from Goddard, E.D.; Hannan, R.B. Polymer/Surfactant Interactions. *J. Am. Oil Chem. Soc*. **1977**, *54*, 561–566. Copyright 1977, John Wiley and Sons [117].

One measure of the chain length influence on PESC formation is the concentration of surfactant required to maintain a precipitated complex with a polyelectrolyte. Goddard et al. examined cationic hydroxyethyl cellulose and various sodium alkyl sulfates with increasing chain lengths and showed that, as the surfactant chain length is increased, the concentration needed to maintain an insoluble complex linearly decreases on a logarithmic scale, highlighting the hydrophobic dependency of the phase behavior of the PESC (Figure 9). Additionally, phase separation is more pronounced for larger surfactant alkyl chain lengths and higher molecular weights [9,13,22]. Furthermore, in studying cationic polymers in combination with a number of anionic surfactants, it was determined that the complex can be re-solubilized at high surfactant concentrations more readily with longer surfactant hydrocarbon chains, more linear chains, and when the ionic head group is at the end of the chain [13,68].

Increasing the molecular weight of a polyelectrolyte has a similar impact on PESC formation as increasing the polyelectrolyte concentration: more surfactant is needed to see transitions at a given concentration [115]. Pojjaźk et al. saw that the precipitation region near stoichiometric match is also broader for low molecular weight samples. Additionally a larger polyelectrolyte to surfactant ratio leads to a smaller hydrodynamic radius for lower molecular weight polyelectrolytes [115], likely because for the same concentration of surfactant, the lower molecular weight polyelectrolyte-surfactant complex is limited in the size that the overall particle can achieve. This is similar to results seen previously by Wang et al. [118]. The hydrodynamic radius is highest near the charge match ratio and where the zeta-potential is close to neutral [115,118,119]. Tseng et al. recently showed that, for PAA and C_14_TAB, no precipitation occurs when the PAA molecular weight is less than 5000 g/mol. At 25,000 g/mol, however, stable particles form, and at 130,000 g/mol and above the particles aggregate and precipitate [80]. The non-cooperative and cooperative binding became more exothermic with increasing molecular weight up to 25,000 g/mol. This is likely due to increasing the number of monomers available for electrostatic and hydrophobic interactions given the same concentration of surfactant. At molecular weights of about 130,000 g/mol and above, the thermodynamic behavior was almost identical for all samples, indicating that the thermodynamics of complexation became independent of the polyelectrolyte molecular weight [80] and the behavior and transitions are driven by the surfactant concentration at high polyelectrolyte molecular weights.

For a PDADMAC and mixed surfactant system (Triton X-100 and SDS), Wang et al. found that the volume fraction of the coacervate was seen to increase with increasing polymer molecular weight [118]. In general, the tendency of a system to phase separate increases with molecular weight [9,46,47,118].

For PESCs formed using block co-polyelectrolytes, changing the molecular weight of the neutral or charged blocks impacts the aggregates formed because it changes the segments available to participate in electrostatic interactions, as well as the segments available to participate in cooperative binding with the surfactant tails. PESCs made from *N*-isopropylacrylamide (PA-*co*-NIPAM (18:82)), which have a large hydrophobic portion, can associate cooperatively with oppositely charged surfactant ions and form soluble complexes beyond the stoichiometric charge match point [68]. This has been seen for a number of systems [18,51,68,82,86,112,120,121] and through simulations [72]. Overall, the presence of blocks results in various PESC structures depending on the hydrophilicity or hydrophobicity of the block. Hydrophilic di-blocks with polyelectrolytes were shown through molecular dynamics simulations to have minimal effect on the final structure of the complex [72,122,123]. However, the inclusion of a hydrophobic block led to the formation of tri-layer core-shell structures. Whereas with tri-block copolymers, the inclusion of hydrophilic blocks led to a “basket structure”, hydrophobic blocks led to a tri-layer core-shell structure, and a hydrophilic and hydrophobic block led to a tadpole-like structure. The presence of hydrophilic and hydrophobic blocks alters the adsorption of surfactant to the polymer chain [72]. It also changes their ability to incorporate hydrophobic co-solutes or be dissolved in solvents [51,121].

#### 3.2.3. Linear Charge Density and Chain Flexibility

Polyelectrolyte charge density determines the number of monomeric units available for electrostatic interaction when the polyelectrolyte is fully charged. The charge density does play a role in the cooperative binding of polyelectrolyte and surfactant systems [78,124,125], but cooperative binding is affected largely by hydrophobic interactions between adjacent surfactant molecules. Therefore, different polyelectrolyte functionalities have larger effects on the CAC and the type of binding than the linear charge density alone [78,91,97].

**Figure 10:**
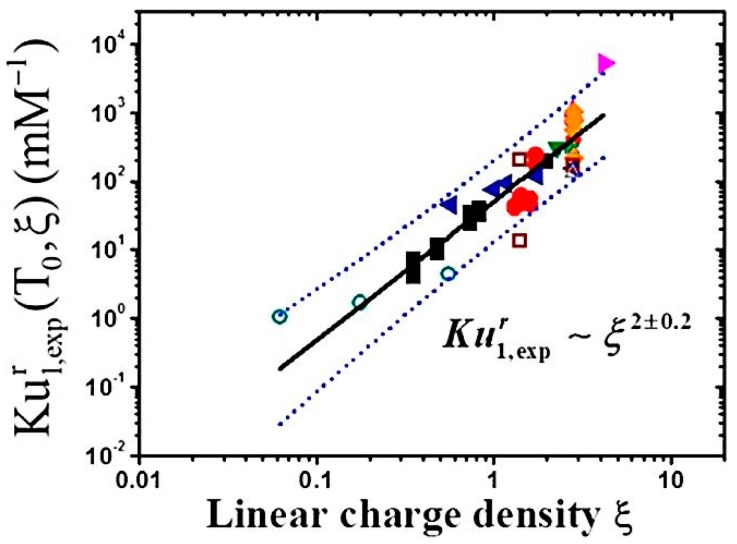
Experimentally reported binding strength data rescaled to SDS reference system as a function of polyelectrolyte reduced linear charge density. Solid black line is power-law regression and blue dotted line is 95% prediction interval. Points represent various studies. Reprinted with permission from Li, D.; Wagner, N.J. Universal binding behavior for ionic alkyl surfactants with oppositely charged polyelectrolytes. *J. Am. Chem. Soc*. **2013**, *135*, 17547–17555, doi:10.1021/ja408587u. Copyright 2013, American Chemical Society [98].

Li and Wagner evaluated the role of charge density and chain hydrophobicity in salt free systems. They derived a rescaled cooperative binding strength (Ku) from the Satake-Yang binding model, which describes the non-cooperative and cooperative binding of surfactant to polyelectrolyte [87], and scaled it by the surfactant micellar free energy to fit experimental data from over 10 different studies. They found that this rescaled binding strength has a squared-power dependence on linear charge density (Figure 10), which means that there is a correlation between increased charge density and cooperative binding strength for many different polyelectrolyte and surfactant systems. This is complementary to Wallin and Linse’s prediction that surfactant binding to a polyelectrolyte chain with higher charge density is more favorable [81]. The Li and Wagner study also noted that the cooperative binding is proportional to polyelectrolyte hydrophobicity, which was not accounted for by Wallin and Linse, as their model (discussed in Section 3.2.2) specifically studied the effect of charge density on polyelectrolyte surfactant systems [24]. 

The charge density directly affects the chain flexibility, with polyelectrolytes with a high charge density typically displaying low flexibility due to the repulsion between charged sites. Increasing the linear charge density or decreasing the flexibility of polyelectrolytes are ways to promote the formation of non-spherical structures of micelles in the presence of polyelectrolytes without salt [50,77]. Goswami et al. used molecular dynamic simulations to study the effect of different PE backbone charge densities on PESC structure and relaxation dynamics. This showed that string-of-pearls/necklace PESCs formed with lower charge density polyelectrolytes and agglomerated core-shell double spherical structures formed when the polyelectrolyte with higher charge density decorated the micelles [109].

It can be difficult experimentally to deconvolute the effects of charge density and inherent flexibility of the polyelectrolyte chain, but simulations can be used to independently change one these parameters while maintaining the other. Monte Carlo simulation studies by Wallin and Linse found that the electrostatic interactions were reduced if the persistence length was high enough compared to the circumference of the surfactant micelle that the chain stiffness reduced how it wrapped around the micelle. If the persistence length of the chain was half of the circumference of the surfactant micelle or greater, then the electrostatic contributions decreased. Therefore the more rigid polyelectrolyte resulted in a higher CAC as compared to a flexible one due to reduced electrostatic interactions [24]. In a follow-up study, while maintaining the flexibility of the polyelectrolyte and varying the spacing between charged moieties, Wallin and Linse found that a flexible polyelectrolyte chain with higher linear charge density allowed the largest decrease of CAC and CMC as compared to a more rigid polyelectrolyte with a lower linear charge density [81]. This is because a higher linear charge density increases the charge of the forming micelle surface, providing more interaction sites and the higher chain flexibility provides more freedom in the conformations that the polymer can adopt to allow complexation with more of those sites. Because this theoretical treatment does not account for hydrophobic interactions, the reduction of CAC in these studies is from electrostatic or rigidity effects alone [24,81].

#### 3.2.4. Effect of pH

The pH of the aqueous system can have a strong effect on the electrostatic interactions of weak polyelectrolytes and charged surfactants, as it dictates whether they behave more neutral or charged. When a weak polyelectrolyte is near its p*K*a, only half of its monomers are charged and can participate in electrostatic interactions. Depending on the polyelectrolyte and solution conditions, it can be highly charged, behave like a neutral polymer, or somewhere in between [53,71,100]. When a polyelectrolyte is highly charged at a given pH and the pH is changed to a point where the polyelectrolyte becomes neutral, the chain transitions from an extended to a coiled state [8,22,37,53]. At pH values where polyelectrolytes are highly charged, non-cooperative and cooperative binding occur at lower surfactant concentrations than when a polyelectrolyte is less charged. This is similar to the effect of decreasing the linear charge density by adding uncharged monomers. As the pH is changed to a point where the polyelectrolyte is highly charged, the CAC also decreases, and the number of aggregates in the PESCs increases [86].

The particle size of solid complexes can also vary with pH. Adjusting the pH to one where the charges on the polyelectrolyte are neutral allows for increased aggregation between particles because charges do not repel each other and the chain is in a more coiled state. The change in pH also causes a shift in structure from a string-of-pearls/necklace conformation to a cylindrical micelle, which also changes the size of the PESC particles, as hydrophobic interactions dominate. This was seen in the case of C_16_TAB and poly 4-vinyl benzoate system (pVB) [100]. Lam et al. found that at a neutral pH (highly charged), the polyelectrolyte-surfactant micelle of (pVB) and C_16_TAB goes from a cylindrical structure to a string-of-pearls structure at low pH where it is near the pKa. The hydrodynamic radius also increases with pH, which is expected for cylinder-to-sphere transitions. The transition from cylindrical to spherical was found to be reversible with pH, which highlights the electrostatic contribution to the system after binding and aggregation has already occurred [100].

Wang et al. studied the thermodynamic association behavior of SDS with PVPNO at various pH values and ionic strengths. PVPNO acts as a polycation at acidic pH and is fully dissociated at a pH of 1.5, but at basic pH of 6 and 8, it is only partially dissociated. At a low pH, electrostatic interactions between SDS and PVPNO are possible, but at high pH the driving force for association is primarily hydrophobic. The effect of charge screening is larger at low pH values when the polyelectrolyte is fully charged [65]. From ITC data, the observed enthalpies of formation at the CAC point of the surfactant with the PVPNO were more exothermic with the change in pH to the highly charged regime for the polyelectrolyte, suggesting that the interaction is in part enthalpically driven and was the most exothermic at a pH where the system is fully charged even without the presence of added salt. The CAC was also the lowest at this pH [65].

**Figure 11:**
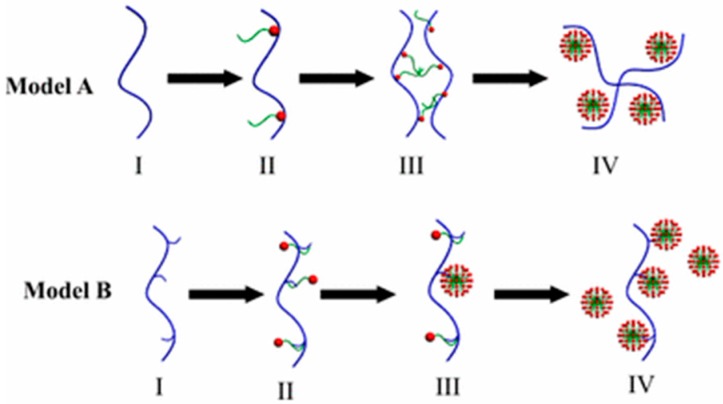
Two proposed mechanisms of the binding interaction between PVPNO and SDS at low (Model A) and high (Model B) pH values. Reprinted with permission from Wang, H.; Fan, Y.; Wang, Y. Thermodynamic association behaviors of sodium dodecyl sulfate (SDS) with poly(4-vinylpyridine *N*-oxide) (PVPNO) at different pH values and ionic strengths. *J. Surfactants Deterg*. **2017**, *20*, 647–657, doi:10.1007/s11743-017-1939-7. Copyright 2017, John Wiley and Sons [65].

Pairing the thermodynamic and turbidity results, Wang et al. present the model shown in Figure 11. Model A depicts the system at a pH where the polyelectrolyte is fully charged and the SDS has a strong impetus to bind through electrostatic interactions with the polyelectrolyte chain. Model B depicts the system at a neutral pH where the polyelectrolyte behaves instead like a neutral polymer and the binding is driven by hydrophobic interactions between the surfactant tail and neutralized polyelectrolyte monomers, leading to formation of a proposed string-of-pearls/necklace structure at higher SDS concentrations where the charged micelles repel one another [65]. These results are similar to work on PEI and SDS at various pH values, where at a low pH PEI is deprotonated and SDS can associate strongly with a combination of electrostatic and hydrophobic interactions, whereas at a neutral pH, hydrophobic interactions dominate [58]. 

#### 3.2.5. Ionic Strength

The binding isotherms for the amount of free surfactant needed to complex a polyelectrolyte shift to the right (higher surfactant concentration) with increasing salt concentration. This is because the higher the salt concentration, the greater the electrostatic charge screening and the more surfactant is needed for complexation [69,77,78,125]. In one case, the addition of NaBr reduced the interaction between sodium carboxymethylcellulose (NaCMC) and mixed Gemini surfactant and neutral TX100 micelles. The polarity in the complexes was shown to decrease in the presence of 0.10 M NaBr. In this case, the salt screened the charges between the polyelectrolyte and surfactants, changing the polarity and allowing for the dominance of hydrophobic interactions [83].

**Figure 12:**
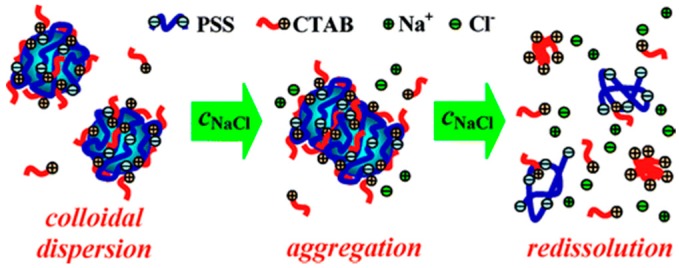
Schematic of effect of salt increase on polyelectrolyte and surfactant association in excess surfactant regime where colloid is charge-stabilized. The blue represents hydrophobic cores. Reprinted with permission from Pojjaźk, K.; Bertalanits, E.; Meźszaźros, R. Effect of salt on the equilibrium and nonequilibrium features of polyelectrolyte/surfactant association. *Langmuir* **2011**, *27*, 9139–9147, doi:10.1021/la2021353. Copyright 2011, American Chemical Society [115].

Mixtures of a cationic surfactant with an anionic polyelectrolyte (PSS and C_16_TAB) and an anionic surfactant with a cationic polyelectrolyte (PEI and SDS) were studied with different salt concentrations. They showed interesting particle aggregation behavior when a high concentration of salt was introduced, as depicted in Figure 12. At low and medium concentrations, salt aids in the aggregation of kinetically trapped colloids, as charges on the surface are neutralized and hydrophobic interactions lead to agglomeration. With a further increase in salt concentration, re-dissolution occurs, as charges are largely screened and aggregates no longer maintain their structure [115]. This is consistent with the Naderi et al. findings, where the turbidity decreased with high salt concentration owning to the re-dissolution of aggregates over time [116].

In some systems of polyelectrolytes with oppositely charged surfactants, the addition of salt leads to the formation of a coacervate phase and the width of the coacervate region in terms of surfactant to polyelectrolyte ratio increases with increasing salt content. A very high ionic strength can further disrupt the coacervate and lead to a re-dissolved single phase, as was described above for precipitated systems [57,119].

#### 3.2.6. Mixing Procedure

The formation of nanoparticles and micelle-like aggregates from PESCs are believed to be kinetically-trapped and not at equilibrium when first made [115,116,126,127], therefore the mixing procedure, adding surfactant to the polyelectrolyte or adding polyelectrolyte to the surfactant, changes the aggregation and colloid size of the resulting complexes. Naderi, et al. showed that when holding the polyelectrolyte poly[2-(propionyloxy) ethyl]trimethylammoniumchloride (PCMA) concentration constant, but varying the SDS surfactant concentration up to the CMC, at low and high surfactant concentrations, the amount of nanoparticles and micelle-like aggregates formed depended on the mixing order [116]. The case where the surfactant was added to the polyelectrolyte had a higher turbidity at low concentrations, while the polyelectrolyte added to the surfactant had a significantly higher turbidity at high concentrations. The behavior is attributed to the initial excess local concentration of either component. Surfactants added to polyelectrolytes can form large charged aggregates early on, whereas the addition of polyelectrolyte to surfactants can form a large network of aggregates. The presence of salt broadens the turbidity peaks for each method due to the screening effect. These effects last beyond 1000 h in extended time studies. This suggests that the choice of procedure traps the formation in a non-equilibrium state [116].

**Figure 13:**
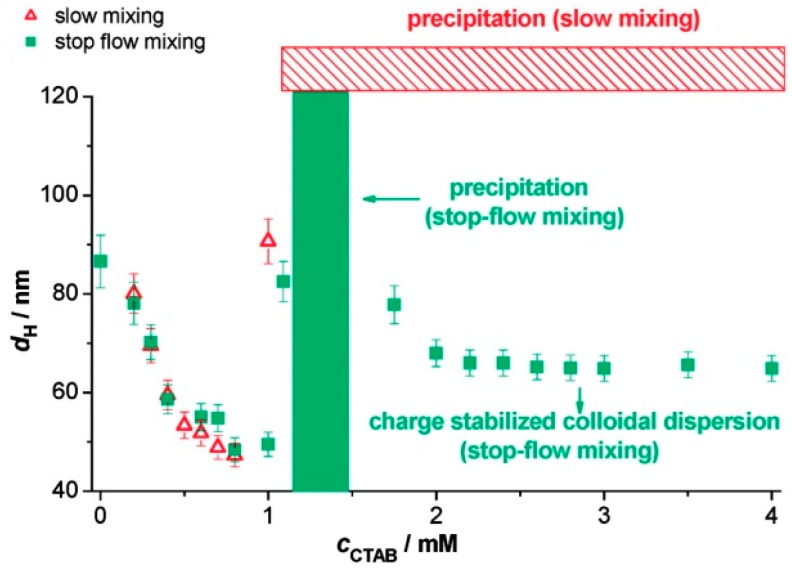
Effect of the applied mixing protocol on the apparent mean hydrodynamic diameter versus surfactant concentration curves, Stop-flow-mixing (green); slow-mixing (red). Reprinted with permission from Pojjaźk, K.; Bertalanits, E.; Meźszaźros, R. Effect of salt on the equilibrium and nonequilibrium features of polyelectrolyte/surfactant association. *Langmuir* **2011**, *27*, 9139–9147, doi:10.1021/la2021353. Copyright 2011, American Chemical Society [115].

The influence of two main mixing methods on the formation of C_12_TAB and PSS complexes were studied: slow mixing, where drop by drop surfactant volumes were slowly added to polyelectrolytes, or stop-flow mixing, where equal volumes of surfactant and polyelectrolyte were added in a stop-flow apparatus which mixed samples within 10 milliseconds. At low surfactant-to-polyelectrolyte ratios, both protocols gave the same results in terms of hydrodynamic radius, except at a small range near charge neutralization where the precipitation occurs at a slightly lower surfactant concentration for the slow-mixing method. At higher surfactant concentrations, the differences are significant, where the stop-flow mixing method resulted in aggregates that were much smaller in size and stable enough to be measure, but macroscale precipitation occurred for the slow-mixing procedure (Figure 13) [115]. They postulate that in the excess surfactant region, for rapid mixing, the aggregation or precipitation of the small PESC nanoparticles formed is hindered by excess surfactant, which results in a sufficient positive surface charge to allow for suspension and prevent macroscopic phase separation. Whereas with the slow-mixing procedure, the particles formed are sufficiently large, that the excess surfactant may not charge the surface enough to prevent sedimentation or precipitation relative to their size. They found that, with the addition of salt, even in the slow-mixing procedure the precipitates were dissolved and the system was transparent throughout the entire surfactant concentration range tested [115]. Mezei et al. found similar results in comparing stop-flow mixing and a slower gentle mixing procedure in terms of colloidal stability at high surfactant concentrations [127].

## 4. Outcomes of Complexation-Rheology

Polyelectrolytes are frequently used as viscosity modifiers. When fully charged, they adopt a rigid rod conformation and when the charges are neutralized with the addition of oppositely charged surfactants, a random coil structure is formed (Figure 2) [8,53]. These changes in conformation result in changes in viscosity, to the degree that even small additions of surfactant result in large increases in the viscosity [128]. In an early study, Fuoss and Strauss studied the effect of added salt on the electrostatic interactions of the polyelectrolytes and the resulting intrinsic viscosity curves. They saw that increasing concentrations of salt drastically decreased the viscosity of poly-4-vinylpyridine (P4VP). With high enough salt, the behavior plateaus and reflects that of a neutral polymer. They interpreted this as a reduction of electrostatic repulsion with salt screening leading to polyelectrolyte collapse [129], which has been seen in other charged systems [130].

For PESCs, increasing the salt concentration allows for increased surfactant aggregation number and higher viscosity and increases the bridging between micelles, in contrast to the effect of salt concentration for pure polyelectrolyte solutions [7,131]. At a high enough concentration of salt, re-dissolution of the PESC complex occurs and the viscosity decreases again [132,133]. With increasing shear rate, polyelectrolyte-micelle solutions behave as Newtonian, shear thickening, and then shear thinning fluids, showing that these solutions are complex fluids and their viscoelastic behavior is shear dependent [132].

**Figure 14:**
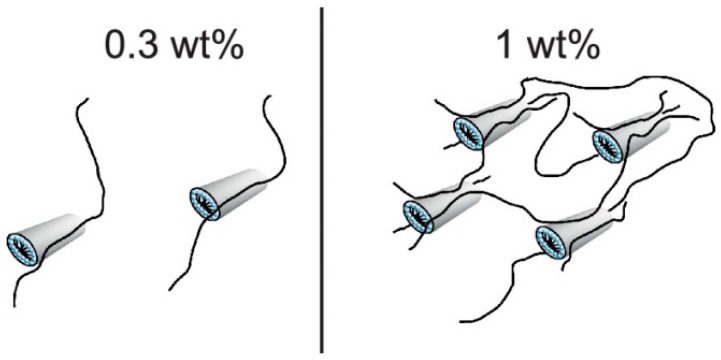
Depiction of Polyelectrolyte-Micelle Structures at low (0.3 wt%) and high (1 wt%) polyelectrolyte concentration showing that cross-links can be achieved at higher concentrations and ultimately higher viscosity. Reprinted from Hoffmann, I.; Farago, B.; Schweins, R.; Falus, P.; Sharp, M.; Prévost, S.; Gradzielski, M. On the mesoscopic origins of high viscosities in some polyelectrolyte-surfactant mixtures. *J. Chem. Phys*. **2015**, *143*, doi:10.1063/1.4928583, with the permission of AIP Publishing [134].

The formation of mixed rod-like polyelectrolyte-surfactant aggregates increases the viscosity for the cationically modified hydroxyethyl cellulose polyelectrolyte JR 400/surfactant system according to Hoffmann et al. [128,133,134]. The viscosity is polyelectrolyte concentration dependent. At high polyelectrolyte concentrations, the viscosity is high due to the interconnectivity between chains from physical crosslinks between PESC aggregates (Figure 14) [128,132,134,135]. The solutions go from a viscoelastic fluid to more elastic as the phase becomes more gel-like [132,135].

**Figure 15:**
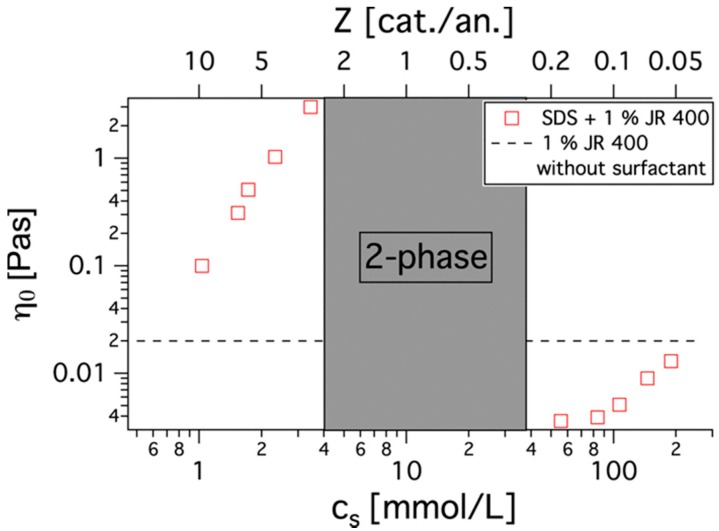
Viscosity of 1 wt% JR 400 polyelectrolyte with increasing concentration of SDS. Z represents the charge ratio between cation and anion. The dashed line is the viscosity of the polyelectrolyte solution alone. Reprinted from Hoffmann, I.; Simon, M.; Farago, B.; Schweins, R.; Falus, P.; Holderer, O.; Gradzielski, M. Structure and dynamics of polyelectrolyte surfactant mixtures under conditions of surfactant excess. *J. Chem. Phys*. **2016**, *145*, doi:10.1063/1.4962581, with the permission of AIP Publishing [133].

At a polyelectrolyte concentration where viscosity is sufficiently enhanced, the viscosity of the solution changes with the stoichiometry between surfactant and polyelectrolyte as shown in Figure 15. As the charge match is approached, the viscosity significantly increases until the two-phase region where precipitates form and come out of solution. After this region, the viscosity drops drastically and increases again slowly, but remains below the viscosity of the polymer alone [133,136]. 

**Figure 16:**
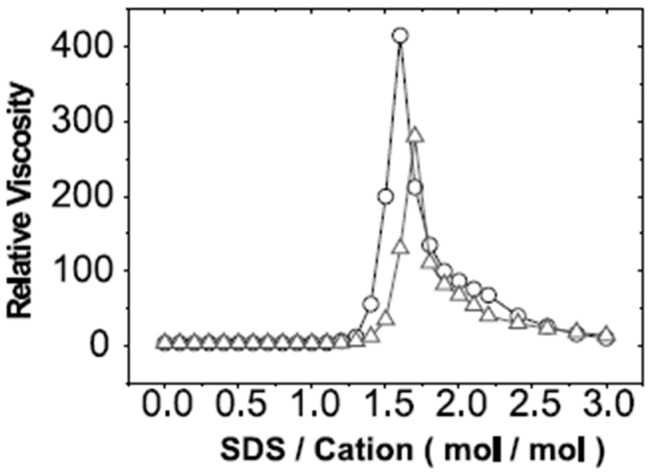
Relative Viscosity for ionene-SDS solutions versus molar ratio. Circle is 6,6-ionene, Diamond is 6,4-ionene. Reprinted from European Polymer Journal, 37, Zheng, X.; Cao, W. Interaction of main chain cationic polyelectrolyte with sodium dodecyl sulfate, 2259–2262, 2001 with permission from Elsevier [137].

The hydrophobic interactions between micelles and a neutralized chain lead to a viscosity enhancement that goes along with phase separation [13] and has a larger role on the rheological behavior than the electrostatic contribution [137]. As seen in Figure 16, a 4,12-ionene and 6,12-ionene showed the highest increase in viscosity at a SDS to polyelectrolyte ratio at charge neutral. However the ratio shifts to the right with the increase in number of carbons in the hydrophobic block for the 6,12-ionene [137]. Wang et al. showed that increasing the alkyl chain groups from 8 to 12 carbon for a Gemini surfactant significantly increased the viscosity, with a significant jump between the CAC and CMC, where the shorter alkyl chain Gemini surfactant steadily increased in viscosity with surfactant concentration during the entire regime [138].

## 5. Conclusions

The binding between polyelectrolytes and surfactants and the structures that form in solution are driven by molecular interactions, particularly the electrostatic and hydrophobic interactions. When the polyelectrolyte is highly charged, the electrostatics typically dominate and the surfactant head attracts to the opposite charges on the polymer. When the polymer is neutral or the conditions are such that the polyelectrolyte has a low charge, the hydrophobic interactions dominate and the surfactant tail attracts to the polymer backbone. The regime of polyelectrolyte charge can be controlled through the molecule design, the pH, and the ionic strength, and these are some of the most powerful parameters to tune to obtain desired structures in the bulk. The concentrations of the polyelectrolyte and surfactant, as well as the ratio between the two, are also critical parameters, both in determining at what concentration the complexes form and in establishing whether they are soluble complexes, small nanoparticles or micelle-like structures, or large precipitates. By modifying the interaction type and strength, as well as the concentrations of the materials, the solution behavior can be controlled and used to adjust the viscosity of mixtures containing PESCs. This approach is highly valuable for a wide variety of industrial formulations, including cosmetics, oil recovery, perfumes and biofuel extraction.
